# The association between language use and food insecurity among Hispanic adults residing in the USA depends on nativity

**DOI:** 10.1017/S1368980023000885

**Published:** 2023-09

**Authors:** Miguel Angel Lopez, Melissa Fuster, Julia M Fleckman, Amy George, M Pia Chaparro

**Affiliations:** 1 Department of Social, Behavioral and Population Sciences, School of Public Health and Tropical Medicine, Tulane University, New Orleans, LA, USA; 2 Department of Spanish and Portuguese, School of Liberal Arts, Tulane University, New Orleans, LA, USA; 3 Nutritional Sciences Program, Department of Health Systems and Population Health, School of Public Health, University of Washington, 305J Raitt Hall, Box 353410, Seattle, WA 98195, USA (current affiliation)

**Keywords:** Food constraint, Food environment, Linguistic isolation, Latino, Linguistic gradient

## Abstract

**Objective:**

To examine the association between language use – predominantly English, English and Spanish equally and predominantly Spanish – and food insecurity among Hispanic adults residing in the USA, 1999–2018.

**Design:**

Pooled cross-sectional study design.

**Setting:**

United States.

**Participants:**

15 073 Hispanic adults.

**Results:**

Compared with Hispanic adults who predominantly spoke English and after adjusting for age, sex, family income-to-poverty ratio, education level and employment status, Hispanic adults who spoke English and Spanish equally (OR = 1·28, 95 % CI = 1·05, 1·56) or predominantly Spanish (OR = 1·25, 95 % CI = 1·04, 1·49) had higher odds of food insecurity. After stratifying by country of birth, language use was associated with higher odds of food insecurity only for Hispanic adults born outside of the USA, but not for Hispanic adults born in the USA. Hispanic adults born outside of the USA who spoke English and Spanish equally (OR = 1·27, 95 % CI = 1·04, 1·55) or spoke predominantly Spanish (OR = 1·24, 95 % CI = 1·04, 1·48) had higher odds of food insecurity when compared with those who predominantly spoke English.

**Conclusion:**

Foreign-born Hispanic adults who speak predominantly Spanish, or English and Spanish equally, have higher odds of food insecurity. Food and nutrition assistance programmes that serve Hispanic immigrants should make sure to provide linguistically and culturally appropriate services to this population.

A household is considered food insecure when it does not have economic and physical access to sufficient, safe and nutrient-dense food that meets dietary needs and food preferences in a socially acceptable manner^(1)^. In 2020, about 38 million (10·5 %) households in the USA were food insecure, with Hispanic households having a disproportionally higher prevalence of food insecurity (17·2 %) when compared with non-Hispanic White households (7·1 %)^(2)^. Among adults, food insecurity is associated with inadequate nutrient intake^(3)^; an increased risk for chronic diseases like obesity^(4,5)^, type II diabetes mellitus^(3)^, hypertension^(3,6)^ and all-cause mortality^(7)^.

At the turn of the 21st century, there were about 44 million Hispanic residents in the USA^(8)^, and it is estimated that 78 % of them spoke predominantly Spanish at home^(9)^. Limited evidence indicates that Spanish-speaking Hispanic individuals have higher rates of food insecurity than those who speak English^(10–12)^. A similar language disparity is also present in research comparing health outcomes among Hispanic individuals with varying levels of language use in the USA^(13)^. This linguistic gradient suggests a pattern where Hispanic individuals who predominantly speak Spanish have worse health outcomes than those who speak Spanish and English equally, and bilingual Hispanic individuals have worse health outcomes than those who predominantly speak English^(13)^. The linguistic gradient has been observed for self-reported physical and mental health^(13–15)^; practicing preventative measures, like undergoing cancer screenings and receiving vaccines^(16,17)^ and being able to afford and schedule healthcare visits^(18)^. Therefore, in respect to language, the degree to which a Hispanic individual experiences negative health outcomes in the USA has more to do with the presence and predominant use of a second language rather than the exclusive absence of English^(13)^.

However, to our knowledge, studies investigating the association between language use and food insecurity have explored language as a binary variable (English proficient *v*. not English proficient or Spanish proficient), and primarily as a proxy for acculturation^(10–12,19)^. The use of language as a proxy of acculturation, or the process individuals experience to adopt a different culture, usually the more dominant one^(9)^, has been heavily criticised in recent years for being linear, static, bidirectional and unethical^(20–23)^, thus suggesting that acculturation is not appropriately captured by language use. In contrast, the present study explores three levels of language use (predominantly Spanish, Spanish and English equally and predominantly English) as an independent risk factor for food insecurity among Hispanic adults living in the USA, under the postulation that existing systematically racist barriers that centre food and nutrition information, assistance and practice around non-Hispanic White’s dominant language and culture may make it difficult for Hispanic adults, particularly predominantly Spanish-speaking Hispanic adults, to procure food and nutrition assistance and services in the USA^(24–28)^. Moreover, this study explores the differences in the proposed association between language use and food insecurity among Hispanic adults by country of birth, as US-born Hispanic adults’ barriers to structurally incorporate into the US food and nutrition environment, which would impact their food insecurity risk, may differ from that of non-US-born Hispanic adults^(2,27)^. In this study’s context, structural incorporation refers to the extent to which a Hispanic individual experiences similar health-related determinants, like food insecurity, to non-Hispanic Whites in the USA. In congruence with the linguistic gradient, the authors hypothesise that Hispanic adults who predominantly speak Spanish will have the highest likelihood of food insecurity, followed by bilingual Hispanic adults and tailed by their predominantly English-speaking counterparts, with these associations being stronger among the foreign-born.

## Methods

### Study design

Data for this cross-sectional study were pooled from the National Health Assessment and Nutrition Examination Survey (NHANES)^(29)^, 1999–2018. NHANES is a nationally representative health and nutrition survey of the noninstitutionalised US’ population that combines in-person interviews with health examinations, such as physical measures and blood draws. NHANES’ study design allows it to collect data across the USA using the same physical instruments across recruitment sites to ensure reliability. NHANES’ in-person interviews include demographic, socio-economic and health- and diet-related questions carried out through stratified and multistage probability sampling^(29)^. While the survey is nationally representative, our analysis was restricted to respondents who self-identified as Hispanic, were at least 18 years of age at the time of the survey, and had all the necessary data to estimate food (in)security. NHANES’ protocol was approved by the National Center for Health Statistics Research Ethics Review Board, and all participants provided informed consent. Additional details are available elsewhere^(29)^.

### Measures

#### Food insecurity

NHANES includes the eighteen-item United States Department of Agriculture’s (USDA) Household Food Security Survey Module (HFSSM)^(30)^, which includes questions about a household’s food access limitations. Responses to the eighteen items are coded as affirmative (a positive response) or dissenting (a negative response). Affirmative responses are summed to calculate a raw score of the scale. Based on the raw scores, households are classified as high food security (0 affirmative responses for households with and without children), marginal food security (1–2 affirmative responses for households with and without children), low food security (3–7 affirmative responses for households with children and 3–5 affirmative responses for households without children) and very low food security (8–18 affirmative responses for households with children and 6–10 affirmative responses for households without children). The present study’s analysis explored the association between language use at home and food insecurity as a collapsed and dichotomised measure, which consisted of food security (high and marginal food security combined) and food insecurity (low food security and very low food security combined).

#### Language use

Language use at home was used to represent respondents’ general language use. NHANES’ response options include only English, more English than Spanish, both equally, more Spanish than English and only Spanish. However, response options for this analysis were collapsed according to the linguistic gradient: predominantly English (only English and more English than Spanish), English and Spanish equally (both equally) and predominantly Spanish (more Spanish than English and only Spanish).

#### Covariates and potential confounders

Demographic covariates included age and gender; potential confounders included family income-to-poverty ratio (IPR), education and employment status. In adjusted models, age and family IPR were each converted into categorical variables of at least four groups to minimise loss of statistical interpretation and improve inference^(31)^. Also, both age and IPR’s maximum limits were set by NHANES and not the authors of this paper. Age was capped at 80 years old and stratified into quintiles: 18–27 (reference), 28–38, 39–50, 51–63 and ≥ 64 years. Family IPR was top coded at 5·00 and classified according to IPR eligibility for federal food assistance programmes^(32)^. Family IPR was calculated as a ratio of monthly family income to the federal poverty level specific to the respondents’ family size. Family IPR was categorised as < 1·00, 1·00–1·30, 1·30–1·85 and > 1·85. Family IPR reference was identified as falling below the federal poverty line for the respective respondent’s survey year, or < 1·00. Sex was categorised as men (reference) and women. Education was categorised as less than a high school degree (reference), high school or General Educational Development equivalent, some college or an Associate in Arts (AA) degree and 4-year college graduate or above. Current employment status was categorised as unemployed (reference), part-time employed (< 40 h per week) and full-time employed (≥ 40 h per week). Further, the respondent’s country of birth was explored as a potential effect modifier. Country of birth was categorised as being born in the USA (i.e. born in the 50 US’ states or Washington D.C.) and elsewhere (including Puerto Rico).

### Statistical analysis

Descriptive statistics of sample characteristics of respondents (*n* 15 073) were reported by the presence of food (in)security. All analyses were carried out using SAS version 9.4 (SAS Institute) and a *P* < 0·05 was used to establish statistical significance in all associations, apart from interaction terms. For interaction terms, a *P* < 0·10 was used to establish statistical significance.

A series of multivariable logistic regression models – crude and adjusted – were used to predict the association between language use at home and food insecurity. Adjusted models controlled for age, gender, education, family IPR and employment. In addition, an adjusted multivariable logistic regression model with an interaction term between language use and country of birth was used to determine if effect modification by country of birth was present. After confirming that the association between a respondent’s language use at home and food insecurity was dependent on their country of birth, adjusted multivariable logistic regression models were stratified by the respondent’s country of birth, with language use at home as the exposure of interest and food insecurity as the outcome. All models included NHANES’ sampling weights and had options specified to control for NHANES’ design effects of stratification, clustering and unequal probability sampling. Standard errors and hypothesis tests adjusted for NHANES’ complex survey design using *proc survey* commands in SAS.

## Results

More than one in four respondents (28·00 %) reported living in food-insecure households. Most of these respondents spoke predominantly Spanish at home (66·91 %) and were born outside of the USA (70·26 %). Further, food insecure respondents were younger, as the majority reported to be between 18 and 27 years old (29·65 %); predominantly women (52·70 %) and unemployed (47·76 %); had less than a high school education (64·23 %) and a family IPR below the federal poverty line (54·09 %). Table 1 presents a more detailed account of the sample’s demographic characteristics by the presence of food (in)security.

**Table 1:** Demographic characteristics of NHANES’ Hispanic respondents by food (in)security, 1999–2018

	NHANES’ Hispanic respondents (1999–2018)			
	Household food security*		Household food insecurity*	
	*n*	%	*n*	%
No. of Respondents	10 843	71·94	4230	28·06
Age, years				
18–27	2556	69·27	1134	30·73
28–38	1930	69·60	843	30·40
39–50	2028	71·06	826	28·94
51–63	1027	73·78	365	26·22
≥ 64	2175	76·80	657	23·20
Sex				
Men	5110	71·86	2001	28·14
Women	5733	72·01	2229	27·99
Country of birth				
United States†	4644	78·70	1257	21·30
Outside of the United States	6192	67·59	2969	32·41
Education				
Less than high school degree	4495	64·90	2431	56·45
High school or General Educational Development equivalent	1915	74·02	672	19·94
Some college or Associates degree	2256	79·77	572	17·82
College graduate or above	1148	91·26	110	5·79
Language spoken at home				
Predominantly English	3627	81·47	825	18·53
English and Spanish, equally	1663	74·54	568	25·46
Predominantly Spanish	5464	65·98	2817	34·02
Family’s income-to-poverty ratio‡				
< 1·00	2280	53·20	2006	46·80
1·00–1·30	1096	65·59	575	34·41
1·30–1·85	1645	73·27	600	26·73
> 1·85	4685	89·87	528	10·13
Employment				
Not employed	4410	68·91	1990	31·09
Part-time employee (< 40 h/week)	1912	71·13	776	28·87
Full-time employee (≥ 40 h/week)	4342	75·61	1401	24·39

* Household food security was estimated with the eighteen-item Household Food Security Survey Module^(30)^. Households with < 3 affirmative responses were classified as food secure and those with ≥ 3 affirmative responses as food insecure.

† United States encompasses the 50 U.S.’ states and Washington D.C. and excludes US territories such as Puerto Rico.

‡ Family IPR was calculated as a ratio of monthly family income to the federal poverty level specific to the respondents’ family size.

The crude and adjusted associations between language use and food insecurity are presented in Table 2. In crude models, those who spoke English and Spanish equally and spoke predominantly Spanish at home were associated with 1·70 and 2·30 higher odds of reporting food insecurity, respectively, when compared with those who spoke predominantly English. In fully adjusted models, the association remained though it was attenuated: Hispanic respondents who both spoke English and Spanish equally or spoke predominantly Spanish had 1·28 and 1·25 higher odds of experiencing food insecurity, respectively, when compared with Hispanic respondents who spoke predominantly English.

**Table 2:** Multivariable logistic regression model between language use at home and food insecurity among NHANES’ Hispanic respondents, 1999–2018

	NHANES’ Hispanic respondents (1999–2018)			
	Crude logistic regression model		Adjusted logistic regression model	
Food insecurity*	OR	95 % CI	OR	95 % CI
Language spoken at home				
Predominantly English	1·00	–	1·00	–
English and Spanish equally	1·70	1·42, 2·03	1·28	1·05, 1·56
Predominantly Spanish	2·30	1·99, 2·66	1·25	1·04, 1·49
Age				
18–27			1·00	–
28–38			1·23	1·05, 1·45
39–50			1·28	1·05, 1·54
51–63			1·09	0·85, 1·40
≥ 64			0·68	0·55, 0·85
Family’s income-to-poverty ratio†				
< 1·00			1·00	–
1·00–1·30			0·63	0·51, 0·78
1·30–1·85			0·43	0·34, 0·53
> 1·85			0·16	0·13, 0·20
Sex, women			0·89	0·81, 0·97
Education				
Less than High School			1·00	–
High school or General Educational Development equivalent			0·88	0·73, 1·05
Some college, or Associates degree			0·76	0·63, 0·92
College graduate or above			0·35	0·26, 0·46
Employment				
Unemployed			1·00	–
Part-time employee (< 40 h/week)			1·04	0·88, 1·24
Full-time employee (≥ 40 h/week)			0·82	0·71, 0·95

* Household food security was estimated with the eighteen-item Household Food Security Survey Module^(30)^. Households with < 3 affirmative responses were classified as food secure and those with ≥ 3 affirmative responses as food insecure.

† Family IPR was calculated as a ratio of monthly family income to the federal poverty level specific to the respondents’ family size.

**Fig. 1:**
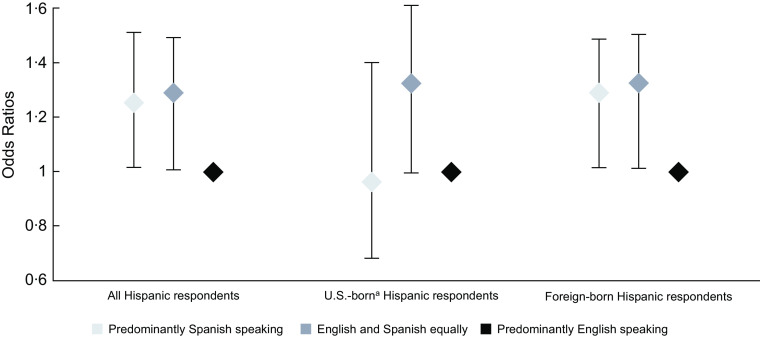
Results of Adjusted Multivariable Logistic Regression Model between Language Use and Food Insecurity, Stratified by Country of Birth, among National Health and Nutrition Examination Surveys’ Hispanic Respondents, 1999–2018. ^a^United States encompasses the 50 US’ states and Washington D.C. and excludes US territories such as Puerto Rico

After confirming that the association between language use and food insecurity was dependent on the country of birth (interaction *P*-values: English and Spanish equally = 0·09; predominantly Spanish < 0·01), we stratified the analysis by respondents’ country of birth (Table 3). Among US-born Hispanic respondents, language use at home was not significantly associated with food insecurity. In contrast, Figure 1 illustrates how foreign-born Hispanic respondents who spoke Spanish and English equally had 1·27 higher odds of reporting food insecurity compared with those who spoke predominantly English at home. Further, foreign-born Hispanic respondents who spoke predominantly Spanish had 1·24 higher odds of experiencing food insecurity when compared with those who predominantly spoke English at home (Fig. 1).

**Table 3:** Adjusted multivariable logistic regression model between language use at home and food insecurity, and stratified by country of birth, among NHANES’ Hispanic respondents, 1999–2018

	NHANES’ Hispanic respondents (1999–2018)			
	U.S.-born Hispanic respondents†		Foreign-born Hispanic respondents	
Food insecurity*	OR	95 % CI	OR	95 % CI
Language spoken at home				
Predominantly English	1·00	–	1·00	–
English and Spanish equally	1·27	0·99, 1·62	1·27	1·04, 1·55
Predominantly Spanish	0·97	0·67, 1·40	1·24	1·04, 1·48
Age				
18–27	1·00	–	1·00	–
28–38	1·15	0·89, 1·49	1·23	1·05, 1·44
39–50	1·45	1·14, 1·84	1·28	1·06, 1·55
51–63	0·77	0·52, 1·13	1·09	0·85, 1·40
≥ 64	0·67	0·50, 0·89	0·68	0·51, 0·77
Family’s income-to-poverty ratio‡				
< 1·00	1·00	–	1·00	–
1·00–1·30	0·83	0·59, 1·16	0·62	0·51, 0·77
1·30–1·85	0·53	0·38, 0·72	0·42	0·34, 0·53
> 1·85	0·16	0·12, 0·21	0·16	0·13, 0·20
Sex, women	0·98	0·82, 1·18	0·88	0·80, 0·97
Education				
Less than High School	1·00	–	1·00	–
High school or General Educational Development equivalent	1·06	0·80, 1·41	0·88	0·73, 1·05
Some college, or Associates degree	0·84	0·62, 1·13	0·76	0·63, 0·92
College graduate or above	0·49	0·32, 0·75	0·34	0·26, 0·46
Employment				
Unemployed	1·00	–	1·00	–
Part-time employee (< 40 h/week	1·12	0·85, 1·48	1·05	0·89, 1·24
Full-time employee (≥ 40 h/week	0·81	0·62, 1·04	0·82	0·71, 0·95

* Household food security was estimated with the eighteen-item Household Food Security Survey Module^(30)^. Households with < 3 affirmative responses were classified as food secure and those with ≥ 3 affirmative responses as food insecure.

† United States encompasses the 50 US’ states and Washington D.C. and excludes US territories such as Puerto Rico.

‡ Family IPR was calculated as a ratio of monthly family income to the federal poverty level specific to the respondents’ family size.

## Discussion

In this study, we used NHANES data to explore the association between the linguistic gradient and food insecurity among Hispanic respondents living in the USA. Through adjusted multivariable logistic regression modelling, we identified that (1) speaking English and Spanish equally or predominantly Spanish was associated with higher odds of food insecurity, but (2) this association was only significant among foreign-born Hispanic respondents once the sample was stratified by country of birth. Those who predominantly spoke Spanish and who spoke English and Spanish equally had a similar likelihood of food insecurity, in conflict with our hypothesis and previous research exploring health and healthcare-related outcomes and the linguistic gradient^(13–18)^.

Prior studies’ findings on language use and food insecurity are similar to the present study: Spanish-speaking Hispanic individuals have higher odds of food insecurity when compared to their English-speaking counterparts^(10–12,19)^. These previous studies present language use as a bivariate exposure, either as English proficiency *v*. not English proficiency^(11)^, or English speaking *v*. Spanish speaking^(10,12,19)^, and do not consider bilingual Hispanic individuals. Doing so limits the interpretability of results as bilingual Hispanic individuals may have unique experiences that differ from predominantly Spanish-speaking and predominantly English-speaking Hispanic individuals^(13)^. It is possible that bilingual Hispanic individuals are able to navigate the US’ food and nutrition environment as well as those who predominantly speak English, but may hold strong cultural ties to their ethnic heritage like those of predominantly Spanish-speaking Hispanic individuals^(33–35)^. Meaning, bilingual Hispanic individuals may be able to translate and understand most food and nutrition information presented to them, but may not culturally identify with that information if not ethnically tailored to the Latin American culture with which they identify^(33–35)^. In practice, this may result in additional barriers a bilingual Hispanic individual must overcome to procure food as well as food and nutrition assistance and services.

Our analysis found similar food insecurity odds among bilingual and predominantly Spanish-speaking Hispanic immigrants. It is possible that both groups experience similar stigma resulting from their accents when speaking English^(36)^, which may place them at a socio-economic disadvantage, affecting their food security. For example, when applying to high-income positions, Spanish-speaking Hispanic adults with noticeable accents were perceived as being less suitable for the job, and fewer were hired when compared to White-presenting English-speaking applicants with no accents^(37)^. Of those that were hired, Spanish-accented Hispanic employees were less likely to be promoted^(37)^. Further, Hispanic individuals born outside of the USA may have more prominent accents when compared with US-born Hispanic individuals, as they may have learned English at an older age or have fewer opportunities to practice, resulting in greater discrimination. Indeed, a study examining foreign accents in English sentences has found that immigrants who lived in the USA longer had less notable accents when compared with more recently arrived immigrants, but more noticeable accents than their US-born counterparts^(38)^. However, not all second-language accents are stigmatised. A similar study found that French-accented applicants were perceived just as favourably, or more, than White-presenting American English-speaking applicants with no accents^(39)^. This linguistic iniquity seems to transcend employment. Low-income Hispanic adults with limited English language skills are less likely to apply for the Supplemental Nutrition Assistance Program (SNAP), the US’ largest federal food safety net, when compared with those who are English proficient. Low-income Hispanic adults with limited English language skills report difficulty understanding current eligibility restrictions, not comprehending changes in eligibility requirements, a lengthy and complex application process and overall lack of federal food assistance programme knowledge^(40)^. Increases in English language skills have been previously associated with greater use of nutrition assistance programs and food security^(41)^. Further, Spanish-speaking Hispanic adults who receive federal food assistance have reported encountering discrimination from store employees when redeeming programme benefits^(42)^, which may reduce future participation.

Previous studies also either limited their sample to Hispanic immigrants^(11)^ or did not account for country of birth in their analysis^(10,12,19)^. However, previous systematic reviews and studies of food insecurity among Hispanic individuals have pointed out that Hispanic immigrants face unique risks of food insecurity when compared with US-born Hispanic individuals^(2,27)^. By not exploring the intricacies behind a respondent’s language use and country of birth, previous studies have restricted the utility of their results as their findings may mask nuances in the association. We found no association between language use and food insecurity for US-born Hispanic adults. In addition to learning English since early childhood, predominantly Spanish-speaking and bilingual US-born Hispanic individuals have distinct circumstances that may result in the lack of observed association between language use and food insecurity. First, US-born Hispanic individuals are US citizens and eligible to apply for federal food assistance programs if they meet other eligibility criteria^(43)^. Second, US-born Hispanic individuals who predominantly speak Spanish, or English and Spanish equally, at home may do so because they are first-generation Americans who live with a predominantly Spanish-speaking family member, like a grandparent; or practice Spanish at home to retain their cultural heritage. Therefore, speaking predominantly Spanish, or Spanish and English equally, at home may be a result of a personal choice rather than a linguistic limitation.

Despite this accumulating body of evidence, no studies to date have applied causal inference to determine if the association between language use and food insecurity is a causal one. Language use is strongly correlated with unmeasured and/or unobserved factors that may influence food insecurity. For example, in the present study, we were unable to account for Hispanic respondents’ immigration status. When compared with an undocumented immigrant, documented immigrants may have greater resources before coming to the USA^(43)^, possibly including the ability to study and practice the English language prior to arrival. Concomitantly, speaking English proficiently, lack of a Spanish accent and being a documented immigrant in the country may provide the Hispanic individual better employment or educational opportunities as well as eligibility to apply for government assistance, like federal food assistance programs^(43)^.

This study has some strengths and limitations. Our ability to explore three levels of language use – predominantly English, English and Spanish equally and predominantly Spanish – among Hispanic adults residing in the USA is a strength. Doing so provided us with a more comprehensive account of how language use is associated with food insecurity among Hispanic individuals and compared it to previous findings concerning the linguistic gradient. Second, accounting for the respondents’ country of birth further revealed nuances in the association not reported in previous studies, to the best of our knowledge. Yet, despite these strengths, the study also has limitations. First, we had to pool almost 20 years of cross-sectional data to achieve the sample size needed to run a stratified analysis. Ensuing studies examining the association between language and food insecurity among Hispanic adults in the USA should investigate the relationship in a longitudinal manner to explore if the observed relationship is stable across time. Second, given the cross-sectional nature of the data, we were not able to identify risk or infer causality of food insecurity and had to limit the interpretability of our results accordingly. We were also constrained by the response options of NHANES’ language items. The authors of this paper recognise that Spanish is not the only language spoken among Hispanic individuals and, given data limitations, we were unable to explore the association between indigenous languages and food insecurity among Hispanic individuals in the USA. Further, despite being used in previous studies^(10,19)^, language use at home may not be representative of an individual’s linguistic abilities and comfort^(44)^. For example, a respondent may live with someone who is more comfortable with Spanish than English (like an immigrant parent) and speak predominantly Spanish at home but speak English in all other spheres of their life. While this has the potential to slightly skew our results, it is likely that it would bring the odds ratios closer to one and dilute the association, as there are fewer scenarios where a Hispanic adult would speak predominantly English at home but Spanish elsewhere. Also, to protect respondents’ privacy, NHANES does not publicly release immigration status of foreign-born Hispanic adults. Therefore, an additional analysis exploring how federal food assistance eligibility and participation may influence the association between language use and food insecurity could not be done. Finally, while prior evidence supports the aetiological explanation hypothesised^(13–28)^, the authors had no concrete variable that allowed us to explore how linguistic discrimination may influence the association between Spanish use and food insecurity in our models. Future research trying to elucidate the association between language and food insecurity should use causal inference methods and examine language proficiency, as opposed to use, while accounting for other established determinants of food insecurity, like (un)documented status and participation in food assistance programs. More research is needed to examine how contextual factors, such as structural racism and food environments, influence the association between language proficiency and food insecurity among Hispanic adults attempting to procure linguistically and culturally appropriate food and nutrition assistance/services in the USA.

## Conclusion

Hispanic individuals are one of the largest and fastest-growing ethnic groups in the USA and continue to face increased rates of food insecurity when compared with non-Hispanic Whites^(2)^, even when traditional risk factors, like socio-economic status, are accounted for^(27)^. In accordance with this expected population growth, it is imperative that public health professionals continue to explore determinants of food insecurity. Our study identified an association between language use, an understudied social determinant of health and food insecurity among Hispanic adults residing in the USA. We did not find support for the linguistic gradient – in which predominantly Spanish speakers would have had greater odds of food insecurity than bilinguals, whom in turn would have had greater odds of food insecurity than predominantly English speakers. Instead, we found that Spanish-speaking and bilingual Hispanic adults had increased odds of food insecurity when compared with their predominantly English-speaking counterparts only if they were born outside of the USA. Based on these findings, public health nutrition programs and policies should focus on removing structural barriers to food access and on providing linguistically and culturally appropriate food and nutrition assistance/services to individuals who need them.
